# Interactions between E-Cadherin and MicroRNA Deregulation in Head and Neck Cancers: The Potential Interplay

**DOI:** 10.1155/2014/126038

**Published:** 2014-08-04

**Authors:** Thian-Sze Wong, Wei Gao, Jimmy Yu-Wai Chan

**Affiliations:** Department of Surgery, The University of Hong Kong, Pokfulam, Hong Kong

## Abstract

E-cadherin expression in the head and neck epithelium is essential for the morphogenesis and homeostasis of epithelial tissues. The cadherin-mediated cell-cell contacts are required for the anchorage-dependent growth of epithelial cells. Further, survival and proliferation require physical tethering created by proper cell-cell adhesion. Otherwise, the squamous epithelial cells will undergo programmed cell death. Head and neck cancers can escape from anoikis and enter into the epithelial-mesenchymal transition stages via the modulation of E-cadherin expression with epigenetic mechanisms. At epigenetic level, gene expression control is not dependent on the DNA sequence. In the context of E-cadherin regulation in head and neck cancers, 2 major mechanisms including *de novo* promoter hypermethylation and microRNA dysregulation are most extensively studied. Both of them control E-cadherin expression at transcription level and subsequently hinder the overall E-cadherin protein level in the head and neck cancer cells. Increasing evidence suggested that microRNA mediated E-cadherin expression in the head and neck cancers by directly/indirectly targeting the transcription suppressors of E-cadherin, ZEB1 and ZEB2.

## 1. Introduction

Head and neck cancers could be developed from multiple sites in the head and neck regions including nasopharynx, oral cavity, oropharynx, larynx, and pharynx. It is the sixth most common cancer worldwide and is the fourth most common cancer in the male population in Europe [1]. Head and neck cancer is a multifactorial disease. Well-known risk factors include tobacco and alcohol consumption [2]. Epidemiology data suggested that there is a significant increase in the risk for cancer development in the group of smokers and drinkers, especially in young adults [3]. In particular geographic regions, the cancers from the oral cavity and oropharyngeal regions are linked with the chewing of tobacco or betel nut [4, 5]. Head and neck regions are subjected to “field cancerization.” Early genetic changes could possibly be reverted if the risk factors are removed in the early stages [6, 7]. Patients with precancerous lesions such as leukoplakia and erythroplakia have a high probability to progress to carcinoma without early diagnosis. Histologically, squamous cell carcinoma is the major clinical presentation of head and neck cancers. The prognosis of head and neck cancer is poor especially when regional migration to cervical lymph nodes and metastasis to distant organs are presented. The migration and invasion of the head and neck cancer are sequential processes. In oral tongue cancer, the risk for lymphatic metastasis increased with the tumor volume [8]. It is evidenced that the tumor depth is a predictive factor for cervical metastasis [9]. Further, the relevance between regional lymph node migration of head and neck cancers and poor prognosis is documented in hypopharyngeal carcinoma patients in which patients with high positive lymph nodes ratio closely tend to have poorer survival [10].

## 2. E-Cadherin Dysregulation in Head and Neck Cancers

Epithelial cadherin (E-cadherin or cadherin 1) is a transmembrane glycoprotein that functions as cell adhesion molecule (CAM). The gene encoding E-cadherin (CDH1) located at chromosome 16q22.1, a common hotspot of genetic abbreviations such as loss of heterozygosity (LOH) and mutations. Structurally, the E-cadherin protein contains five extracellular cadherin repeats, a transmembrane region, and a highly conserved cytoplasmic tail. It is a calcium-dependent molecule involved in cell-cell adhesion, cell dissociation, and cell motility. The transmembrane glycoprotein establishes homophilic interactions with adjacent E-cadherin molecules forming the epithelial junctional complexes, which mediate the cell-cell adhesion in the epithelial tissues [11]. On the other hand, the cytoplasmic tail forms a link with the actin cytoskeleton of the cells [12].

E-cadherin expression is ubiquitous in normal stratified squamous epithelium of the oral cavity and oropharynx. E-cadherin suppressed head and neck epithelial cancer cells escape from contact-dependent growth and develop migratory phenotype with low differentiation stage, suggesting that E-cadherin has the potential to contribute to the transformation steps. It has been hypothesized that the E-cadherin negative cells are dissociated at the invasive fronts at the late progressive stages of oral tongue cancer development resulting in the metastasis to the regional lymph nodes [13]. In the* in vitro* model, cell lines expressing E-cadherin will appear as cuboidal morphology in well-differentiated cell lines and formed cobblestone colonies. In poorly differentiated cell lines, the cells will be shown as spindle-shaped with weak or no E-cadherin expression [14].

E-cadherin expression level is associated with the behavior of the cancers cells in the animal models and usually accompanied with high invasion ability. Cell lines losing E-cadherin expression displayed an increase in the invasive capacity. The oral tongue cancer cell line treated with human monoclonal antibodies will form highly invasive clones [13]. Similar to the cell line models, E-cadherin expression in the primary oral tongue cancer tissue is correlated with the cancer grading and is an indicator of poor prognosis for oral tongue cancer patients [15]. Further, low E-cadherin expression is an indicator of late cervical lymph node metastasis and is a poor prognostic factor for the overall survival of oral squamous cell carcinoma patients [16, 17]. Accumulating data on clinical specimens provided mounting evidence for the use of E-cadherin as an independent risk factor for oral tongue cancer patients [17]. The loss of homophilic cell-cell adhesive property in the E-cadherin suppressed cells will lead to the development of invasive phenotype. If the loss is a consequence of epigenetic aberration, the acquired invasive phenotype could possibly be reversed with the use of epigenetic drugs [18].

## 3. Epithelial-Mesenchymal Transition (EMT) and E-Cadherin

Epithelial head and neck cancer cells could acquire the ability to migrate and invade the surrounding tissues. As the cells move into the lymphatic system, they could be detected in the regional lymph nodes via route of peripheral lymphovascular channels. This process is generally regarded as epithelial-mesenchymal transition (EMT) accompanied by the conversion of highly aggregated epithelial cells to migratory and invasive cells. In the EMT conversion, E-cadherin is suggested to be an important player due to its functions in homophilic interaction with the adjacent cells and the observation that its expression is frequently lost in epithelial cancers with the migratory phenotype [19]. Epithelial cells are anchorage-dependent cells in which the growth and survival are dependent on extracellular matrix adhesion. When the normal epithelial cell loses cellular contact with the ligands in the extracellular matrix, it will undergo a specific type of programmed cell death (i.e., anoikis) triggered by multiple protein kinases/phosphatase including phosphoinositide-3 kinase (PI3K), extracellular signal-regulated kinase (ERK), and Jun N-terminal kinases (JNKs) [20]. Epithelial cancer cell undergoing EMT will acquire a fibroblast-like morphology with a high motile phenotype [21]. Apart from physical alterations, E-cadherin could induce EMT by induction of transcription factor such as Twist [21]. Twist is usually associated with the advanced cancers and linked with the positive lymph node, lung metastasis, and poor survival of oral squamous cell carcinoma patients [22].

## 4. Epigenetic Control of Gene Expression

Epigenetic regulation refers to control of gene expression without the reliance on DNA sequence [23]. The most common form of epigenetic regulation includes changes in the CpG island methylation patterns in the promoter region via addition of an aberrant methyl group to the cytosine residue, remodeling of the chromatin structure via modifying the histone protein core, and control of the expression level with the negative transcription regulator, microRNA. In terms of the epigenetic control of E-cadherin expression in the head and neck cancers, the most frequently reported mechanism includes promoter DNA hypermethylation and overexpression of the target microRNA.

## 5. Epigenetic Control of E-Cadherin Expression by DNA Methylation

Germline and somatic mutation of E-cadherin gene has been found in diffuse-type gastric cancer, colorectal cancer, lobular and invasive ductal breast cancer, endometrial ovarian cancer, and prostate cancers [12]. Although loss of E-cadherin expression is also common in head and neck cancer, especially in the metastatic tumors, genetic abbreviation in head and neck cancers is relatively rare. In contrast, the most common form of E-cadherin alterations in head and neck cancer is DNA methylation.

DNA methylation refers to the covalent addition of a methyl group to the cytosine residue in the CpG dinucleotide [24]. Methyl CpG dinucleotide in fact is distributed throughout the normal genome. In cancer, however, methyl CpG cluster (CpG islands) embedded in the regulatory regions of the tumor-related genes becomes methylated by the* de novo* DNA methyltransferases (DNMTs). The dense methylation incidence in the CpG islands is referred to as DNA hypermethylation which will lead to transcriptional silencing of the associated genes [25, 26]. Methylated DNA sequence will allow the preferential binding of methylated DNA binding proteins (e.g., MeCP2 and MBD2) and histone deacetylases leading to the nucleosome remodeling and histone modification and thereby hindering the accessibility of the transcription machinery [23].

## 6. Promoter Hypermethylation of E-Cadherin

**Figure 1:**
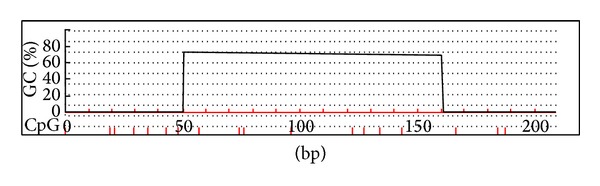
E-cadherin promoter region contains dense CpG dinucleotide density.

Promoter hypermethylation is a potential epigenetic mechanism to the E-cadherin gene due to the dense CpG dinucleotide density in the transcription regulatory region (Figure 1). In cancer cell lines which grew as monolayer, treatment of the E-cadherin methylated cell lines with demethylating agent such as 5-azacytidine can restore the transcription and reverse the invasive phenotype [18]. However, the reversion with demethylation is not effective in all cases suggesting that other mechanisms exist in silencing E-cadherin at transcription level in the head and neck cancer cells [27]. Methylation of 5′ promoter region of the E-cadherin is found to be an early event in oral cancer lesions [28]. The presence of E-cadherin methylation is independent of the invasive and metastatic potential of the cancer cells [29]. At first, methylated DNA is thought as a specific cancer marker as the* de novo* methylated sequence is found largely in the cancerous tissue. In addition, the methylated E-cadherin DNA is present in the peripheral blood and oral rinse of head and neck cancer patients leading to the suggestion of using it as an noninvasive diagnostic marker in head and neck cancer detection. This potential use has also been explored in the oral rinse collected from oral squamous cell carcinoma patients [30]. However, before moving to the clinical use, it should be considered that methylated E-cadherin could possibly be deposited in the noncancerous epithelia in the head and neck region. Using pyrosequencing, the existence of methylated E-cadherin DNA is demonstrated in both oral squamous cell carcinoma and normal tissues obtained from the resection margin of the same patients with no statistical difference [31].

## 7. E-Cadherin Hypermethylation Induced by Human Papilloma Virus (HPV)

*De novo* methylation of E-cadherin DNA could be induced by external factors such as virus infection. HPV infection is particularly common in oropharyngeal carcinoma and is considered as an independent risk factor [32, 33]. HPV positive tumor demonstrated characteristic features in epidemiology, clinical behavior, and molecular presentation and is regarded as a distinctive entity in head and neck cancer management [34, 35]. In the context of DNA methylation patterns, head and neck cancer infected with HPV has distinctive methylation imprints in comparison with the HPV negative counterparts [36]. The corresponding methylation pattern mediated by HPV infection is sometime referred to as methylation signature or methylator phenotype of head and neck cancers, in which high frequency of E-cadherin DNA methylation is observed and supports the idea that HPV facilitates E-cadherin suppression via altering the methylated CpG imprinting [36].

## 8. Epigenetic Controls of Gene Expression with MicroRNA

MicroRNA is a group of highly conserved noncoding RNA molecules with size that usually ranged from 20 to 22 b.p. long [37]. It was first identified in* Caenorhabditis elegans* (*C. elegans*) and is now recognized as a kind of epigenetic regulator in cell growth, proliferation, morphogenesis, tissue remodeling, and development. Precursor microRNA is transcribed by RNA polymerase II and modified by the RNase III-type nuclease, Dorsha, in the nucleus to form the primary microRNA. Primary microRNA will later be transported into the cytoplasm by Exportin-5 (a Ras-GTP-dependent dsRNA-binding protein) and cleaved by Dicer (RNase complex) and TRBP [TAR (transactivation-responsive RNA of HIV-1) RNA-binding protein] to generate the mature microRNA. Mature microRNA functions as translation inhibitor by hindering the translation machinery or promoting the recruitment of Argonaute-containing RNA-induced silencing (RISC) complex after binding to the target mRNA via a partial/incomplete complementary binding. The matching between the seed sequence (2–7 b.p.) of the microRNA with the target mRNA sequence would suffice to initiate the regulation cascade by microRNA [38]. MicroRNA expression patterns are tissue-specific. Increasing evidence suggested that head and neck cancers have a distinctive microRNA expression pattern which account for the characteristic clinical features of head and neck cancers [39]. Theoretically, the microRNA profile is highly correlated with the gene expression patterns displayed by the head and neck cancer cells as microRNA could directly control the translation of their target mRNA transcripts leading to the reduction in protein expression [39].

## 9. Potential Linkage between E-Cadherin and MicroRNA Networks

**Figure 2:**
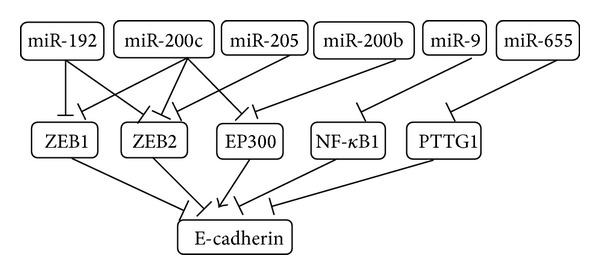
MicroRNAs regulate the expression of E-cadherin by targeting multiple transcription regulators.

In comparison with DNA methylation which modify the E-cadherin gene sequence directly, microRNAs regulated the expression of E-cadherin by targeting different transcription or transcription-associated factors linking with the transcriptional processes including NF-*κ*B1, ZEB1, ZEB2, EP300, and PTTG1 (Figure 2).

### 9.1. MicroRNA-9

MicroRNA-9 expression is activated by the MYC/MYCN signaling cascade and triggers EMT by targeting E-cadherin mRNA [40]. In c-myc-induced mouse mammary tumors, a significant increase in microRNA-9 is observed [40]. MicroRNA-9 is classified as tumor suppressing microRNA, as reduction in microRNA-9 expression by DNA methylation is found in the oral, oropharyngeal, and laryngeal carcinoma [41, 42]. Overexpressing microRNA-9 in the aggressive oral squamous cell carcinoma cell lines suppresses the invasion ability [42]. The microRNA-9 contains the seed sequence targeting the 3′-untranslated region (UTR) of NF-*κ*B1 and therefore suppressed the function of E-cadherin repressor, Snail1 [43].

### 9.2. MicroRNA-10b

Distinct from other head and neck cancers, nasopharyngeal carcinoma is consistently associated with Epstein-Barr virus (EBV) infection. EBV is found nearly in all the World Health Organization (WHO) type II and type III nasopharyngeal carcinomas. E-cadherin expression in nasopharyngeal carcinoma is controlled by Twist (a highly conserved transcription factor of the basic helix-loop-helix protein) via the Wnt signaling cascade [44]. Twist is involved in the invasion and metastasis of nasopharyngeal carcinoma and is closely correlated with the regional lymph node status of the nasopharyngeal carcinoma patients [45]. The increase in Twist expression is induced by one of the viral oncoprotein, latent membrane protein 1 (LMP1), in the cancer cells. Recently, it was noticed that Twist also controls E-cadherin by inducing microRNA-10b expression [46]. NPC cells overexpressing microRNA-10b demonstrated lower E-cadherin expression with higher mobility [47]. The migration inducing effects is demonstrated in the mouse bone marrow-derived mesenchymal stem cells with microRNA-10b [48]. In addition, the viral protein LMP1 could also be a modulator of microRNA-10b in the nasopharyngeal carcinoma cells [49].

### 9.3. MicroRNA-31

Compared with the normal oral mucosa, high microRNA-31 expression is found in the oral potential malignant disorder (OPMAD) tissues suggesting that microRNA-31 is a candidate oncogenic microRNA involved in the early development of oral tongue cancers [50]. Ectopic expression of microRNA-31 could promote the growth and immortalization of the nontumorigenic oral keratinocytes [50]. In a three-dimensional organoid culture derived from colon carcinoma, increase in microRNA-31 expression is observed during EMT induced by transforming growth factor *β*, a proinvasion/metastasis inducer [51]. Treatment of the cancer cells with transforming growth factor *β* could induce E-cadherin redistribution from the cell surface to cytoplasm without affecting the expression level [51]. In oral mucosa, E-cadherin redistribution from membrane to the cytoplasm is observed during EMT [52].

### 9.4. MicroRNA-192

MicroRNA-192 is overexpressed in the supraglottic laryngeal cancer patients with cervical lymph node metastasis [53]. Overexpression of miR-192 in the proximal tubular cells could suppress the expression of both the 2 major E-cadherin suppressors: ZEB1 (TCF8/deltaEF1) and ZEB2 (SMAD-interacting protein 1 [SIP1]/ZFXH1B) [54]. They are E-box binding transcription factors associated with the EMT initiation in epithelial cells by repressing epithelial differentiation and cell-cell adhesion [55, 56]. The inverse correlation of ZEB1 with E-cadherin leads to the loss of intercellular adhesion. Further, it is suggested that ZEB1 is linked with the cancer cell dedifferentiation and cell polarity [55].

### 9.5. MicroRNA-200 Family

The microRNA-200 family consists of 5 members: microRNA-141, microRNA-200a, microRNA-200b, microRNA-200c, and microRNA-429. MicroRNA-200 family is associated with the invasive phenotype of multiple human cancers [57]. In oral tongue squamous cell carcinoma, expression of the microRNA-200 family members is controlled by DNA methylation. Activation of microRNA-200a, microRNA-200b, microRNA-429, and microRNA-200c is closely correlated with the global CpG methylation reduction. It is suggested that miR-200 family controls E-cadherin at transcription level by targeting the E-cadherin E-box repressors ZEB1 and ZEB2 [58, 59]. In the mouse model, microRNA-200c is a positive regulator of E-cadherin. MicroRNA-200c can induce E-cadherin expression [60]. Reversely, deletion of microRNA-200c results in E-cadherin downregulation [60]. Apart from the ZEB, microRNA-200 could also regulate E-cadherin expression by targeting the transcription coactivator of E-cadherin, EP300 [61, 62]. The E1A-associated cellular p300 is a positive E-cadherin regulator protein and functions by binding to the two p300 binding sites located within the E-cadherin regulatory sequence [63]. Of the 5 miR-200 family members, microRNA-200b and microRNA-200c are suggested to be the regulators of E-cadherin through targeting p300 transcripts in the cancer cells [61].

### 9.6. MicroRNA-205

MicroRNA-205 is expressed at a high level in normal tissue containing squamous epithelium [64]. In head and neck squamous carcinoma, high microRNA-205 is also observed, especially in case when positive lymph node is detected [64]. In contrast, in laryngeal squamous cell carcinoma, microRNA-205 is significantly downregulated in the primary cancer tissues and cell line [65]. MicroRNA-205 could target ZEB2 mRNA and hence repress E-cadherin expression in the cancer cells [66].

### 9.7. MicroRNA-655

Apart from microRNA-200 family, the E-cadherin suppressor ZEB1 is controlled by another EMT-related microRNA, microRNA-655. Reduction of microRNA-655 expression has recently been reported in the oral squamous cell carcinoma [67]. Overexpressing microRNA-655 could suppress the invasion and migration of the cancer cell lines together with the induction of E-cadherin expression [67]. In esophageal squamous cell carcinoma, microRNA-655 could inhibit tumor cells migration via targeting pituitary tumor-transforming gene-1 (PTTG1), an invasion and migration promoter. In breast cancer cells, PTGG1-overexpressing cells have spindle shape with reduction in E-cadherin expression [68]. In addition, expression levels of EMT markers (including N-cadherin and vimentin) were induced in the PTGG1-expressing cells.

## 10. Cancer Cells Interact with the Environmental Perturbation by Regulating Their MicroRNA Expression Patterns: The Role of Hypoxia-Related MicroRNA

Intratumoral hypoxia is a result from the poor vasculature formed in the rapid proliferating cancer leading to the development of a microenvironment with low oxygen environment. The abnormality in the tumor microvasculature will increase the diffusion distances leading to the disturbance of gas exchange. Consequently, the oxygen transport capacity of the blood in the tumor bulk will be reduced as the tumor grows [69]. With the deteriorating gas exchange condition, normal tissues will be subjected to cellular quiescence, differentiation, and apoptosis leading to the impairment of growth and development [70]. In order to survive, cancer cells must tolerate the oxygen-deprived environment by switching their gene expression patterns to adapt the anaerobic condition [71]. Tumor cells with the ability to survive and adapt under the adverse condition are selected through clonal expansion with aggravate aggressiveness and genomic changes [69, 70].

Under hypoxic condition, one hallmark feature of head and neck cancer is the increase in invasiveness with enhancement of EMT ability with altering E-cadherin expression patterns [72]. It is suggested that HIF-1 could regulate E-cadherin indirectly by controlling the expression of E-cadherin regulators [72, 73]. With our understanding of the microRNA regulatory mechanism, it is now recognized that this is achieved by hypoxia-inducible factor-1 (HIF-1), which modulates the microRNA production machinery subsequently in the cancer cells in response to the low-oxygen microenvironment. In view of the emerging role of microRNA in gene regulation, several groups suggested that the hypoxic condition or HIF-1 itself could function as a microRNA regulator in controlling specific microRNA expression under the adverse condition. For example, microRNA-210, a tumor suppressing microRNA in head and neck cancers, is suggested to be a responsive element to the hypoxic stress and the expression is controlled by HIF-1 [74]. As E-cadherin expression is also controlled by HIF-1 under hypoxic condition, it remains to be explored whether HIF-1 could also control E-cadherin by inducing specific microRNA expression. Recent data suggested that microRNA could be the upstream regulator of HIF-1. MicroRNA-31, for instance, can regulate HIF-1 expression in head and neck squamous cell carcinoma by controlling its regulator factor-inhibiting hypoxia-inducible factor (FIH) under normoxic condition [75]. Another example is the E1A-associated cellular p300 transcriptional coactivator (EP300). As mentioned above, the transcription coactivator EP300 (regulated by the miR-200 family) is an inducer of E-cadherin expression on the one hand. On the other hand, it is also a transcription activator of HIF-1A (hypoxia-inducible factor-1 alpha). This has led to the suggestion that the HIF-1A and microRNA-210 family are intimately linked and cooperated in controlling E-cadherin expression under hypoxic condition.

## 11. Conclusions

E-cadherin expression in the head and neck epithelium is essential for the morphogenesis and homeostasis of epithelial tissues. Reduced E-cadherin expression has been observed in head and neck cell lines, animal xenograft model, and primary tumor of head and neck cancer. Decreased E-cadherin expression in the primary tumor is linked with the invasiveness of the cancers cells. Low E-cadherin expression is associated with poor prognostic factor of overall survival and late cervical metastasis in head and neck cancer patients. E-cadherin is suggested to be an important player in EMT, a process in which epithelial head and neck cancer cells acquire the ability to migrate and invade the surrounding tissues. The expression of E-cadherin was under epigenetic controls: promoter hypermethylation and microRNA. Environmental perturbation such as hypoxic condition and HPV infection could also regulate the expression of E-cadherin via affecting hypoxia-related microRNA or promoter methylation. Various microRNAs regulated the expression of E-cadherin by targeting genes involved in the transcription process including NF-*κ*B1, ZEB1, ZEB2, EP300, and PTTG1. In light of the regulatory network between microRNAs and E-cadherin, it is feasible to reintroduce the expression of E-cadherin via microRNA-based therapy aiming at controlling the regional and distant metastasis of head and neck cancers. A more comprehensive understanding of the regulatory mechanisms is required for the clinical use of microRNAs to modulate E-cadherin expression in the clinical settings.
